# 25-hydroxycholecalciferol reverses heat induced alterations in bone quality in finisher broilers associated with effects on intestinal integrity and inflammation

**DOI:** 10.1186/s40104-021-00627-6

**Published:** 2021-10-08

**Authors:** Huaiyong Zhang, Maryam Majdeddin, Djoere Gaublomme, Bernard Taminiau, Matthieu Boone, Dirk Elewaut, George Daube, Iván Josipovic, Keying Zhang, Joris Michiels

**Affiliations:** 1 Laboratory for Animal Nutrition and Animal Product Quality, Department of Animal Sciences and Aquatic Ecology, Ghent University, 9000 Ghent, Belgium; 2 Key laboratory of Animal Disease-resistant Nutrition, Ministry of Education, Institute of Animal Nutrition, Sichuan Agricultural University, Ya’an, 611130 Sichuan China; 3 Unit Molecular Immunology and Inflammation, VIB Center for Inflammation Research, Ghent University and Department of Rheumatology, Ghent University Hospital, 9000 Ghent, Belgium; 4 Department of Food Sciences – Microbiology, University of Liège, 4000 Liège, Belgium; 5 Ghent University Centre for X-ray Tomography (UGCT), Ghent University, 9000 Ghent, Belgium; 6 Department of Physics and Astronomy, Radiation Physics Research Group, Ghent University, 9000 Ghent, Belgium

**Keywords:** Bone remodeling, HS, Inflammation, Intestinal barrier, Tibial mass

## Abstract

**Background:**

Alterations in ambient temperature have been associated with multiple detrimental effects on broilers such as intestinal barrier disruption and dysbiosis resulting in systemic inflammation. Inflammation and 25-hydroxycholecalciferol (25-OH-D_3_) have shown to play a negative and positive role, respectively, in the regulation of bone mass. Hence the potential of 25-OH-D_3_ in alleviating heat induced bone alterations and its mechanisms was studied.

**Results:**

Heat stress (HS) directly induced a decrease in tibia material properties and bone mass, as demonstrated by lower mineral content, and HS caused a notable increase in intestinal permeability. Treatment with dietary 25-OH-D_3_ reversed the HS-induced bone loss and barrier leak. Broilers suffering from HS exhibited dysbiosis and increased expression of inflammatory cytokines in the ileum and bone marrow, as well as increased osteoclast number and activity. The changes were prevented by dietary 25-OH-D_3_ administration. Specifically, dietary 25-OH-D_3_ addition decreased abundance of B- and T-cells in blood, and the expression of inflammatory cytokines, especially TNF-α, in both the ileum and bone marrow, but did not alter the diversity and population or composition of major bacterial phyla. With regard to bone remodeling, dietary 25-OH-D_3_ supplementation was linked to a decrease in serum C-terminal cross-linked telopeptide of type I collagen reflecting bone resorption and a concomitant decrement in osteoclast-specific marker genes expression (e.g. cathepsin K), whereas it did not apparently change serum bone formation markers during HS.

**Conclusions:**

These data underscore the damage of HS to intestinal integrity and bone health, as well as that dietary 25-OH-D_3_ supplementation was identified as a potential therapy for preventing these adverse effects.

**Supplementary Information:**

The online version contains supplementary material available at 10.1186/s40104-021-00627-6.

## Introduction

Heat stress (HS) is well documented to have a negative influence on livestock productivity and climate change may exacerbate the incidence of HS. Fast-growing modern broilers with intensive metabolic heat production, covering feathers and lack of sweat glands are particularly prone to HS due to limited capability for heat dissipation [1]. One of the most noticeable developmental problems associated with HS in all poultry is a pronounced induction of leg abnormalities, as shown in broilers [2, 3], laying hens [4], and turkeys [5]. Consequently, the incidence of leg disorders, such as lameness, causes economic losses to the producer [6], and this is also a welfare issue, resulting from pain and modified behavior [7].

The aetiology of gait problems in poultry induced by HS is varied and complex. An obvious cause of that reduction in bone quality and mass is associated with deficient calcium (Ca) consumption as HS reduces feed intake (FI) and nutrient absorption [8]. Further, elevated temperatures impair gut integrity, thereby increasing systemic inflammation that elicits osteoclastic bone resorption [9]. It has been reported that HS directly affects gut integrity and induces the activation of the innate immune system resulting in systemic inflammation in bovine [10], rats [11], and laying hens [12]. Moreover, heat tends to alter the gut microbiome of caged laying hens [12] and broilers [13, 14]. In mice, using standard tools for manipulation of the gut microbiota, i.e. germ-free and oral antibiotics or probiotics, to alter the microbiota composition are related to changes in bone development and remodeling, as well as changes in bone mechanical strength through actions on the immune system, endocrine system and Ca absorption [9, 15]. For instance, by comparing germ-free mice with conventionally raised mice it was shown that the presence of microbiota led to lower trabecular and cortical bone mass [16]. A further alteration in gut microbiota via oral antibiotics has been reported to affect bone density in mice [17]. Meanwhile, germ-free mice were showing a reduced number of osteoclasts and lower level of Interleukin (IL)-6, receptor activator of nuclear factor-κ B ligand (RANKL), tumor necrosis factor alpha (TNF-α), and CD4^+^T cells in bone [18, 19]. These features were normalized by colonization with gut microbiota from conventionally raised mice [19]. This suggests that the interaction between gut microbiota and the immune system may play a significant role in bone metabolism [9]. In addition, excess glucocorticoids [20] and/or reactive oxygen substances [21] evoked by elevated temperatures interfered with bone formation and resorption, contributing to skeletal damage. However, direct evidences of HS modifying bone health, especially bone remodeling, in broilers are largely lacking.

Several nutritional strategies such as supplementation of diets with tomato pomace and symbiotic product have been used to attenuate the negative effects of HS on bone health in broilers [2, 3]. Vitamin D, a lipid-soluble vitamin, is proposed to improve the walking ability and bone quality, and consequently decrease leg diseases in broilers [22, 23]. Its deficiency can lead to a higher incidence of leg problems in birds [24]. In addition to stimulation of Ca and phosphate (P) absorption in the intestine and renal reabsorption [25], and direct regulation of bone formation and resorption [26, 27], the beneficial effects that vitamin D in bone metabolism may exert may also be related to its many other biological effects, including interference with gut microbiota, and antioxidant and anti-inflammatory properties [28, 29]. All these factors may contribute to bone metabolism [9, 15, 30]. For instance, the administration of vitamin D was noticed to abolish the effects of lipopolysaccharides (LPS) by restoring the expression of zonula occludens (ZO)-1 and claudin-2 [31], and had a protective effect against pepsin-trypsin-resistant gliadin induced tight junction injuries both *in vitro* and *in vivo* [32]. Vitamin D_3_ is also associated with the changes in the composition of gut microbiota, most likely by regulating immune and inflammatory responses [28]. Binding of the active form of vitamin D_3_ was found to induce production of antimicrobial peptides by macrophages, resulting in a selective killing of pathogenic bacteria and increasing the opportunity for colonization with “beneficial” bacteria [33]. A recent review outlines that vitamin D_3_ could promote an increase in the activities of the antioxidant enzymes including superoxide dismutase (SOD), glutathione peroxidase (GSH-Px) and catalase in clinical trials [29], and a decrease in reactive oxygen species (ROS) propagation of monocytes [34]. Based on above findings, we, therefore, hypothesized that dietary vitamin D_3_ supplementation could reverse HS-induced bone loss in finishing broilers by a complex interaction of different mechanisms.

In the present study, we firstly confirmed the HS model in broiler during the finisher phase. HS was induced by a cycling heat episode at 34 °C for 7 h daily for 17 d, opposed to the thermoneutral condition at 22 °C as in the control group. We elucidated the responses in bone quality and intestinal barrier to this long-term HS. Subsequently, 25-hydroxycholecalciferol (25-OH-D_3_), a vitamin D_3_ metabolite, was chosen based on the fact that 25-OH-D_3_ is approximately twice as active as cholecalciferol (vitamin D_3_) in promoting bone strength [35] and thus we evaluated the effects of 25-OH-D_3_ on similar endpoints in broilers subjected to HS.

## Materials and methods

### Birds and study design

**Fig. 1:**
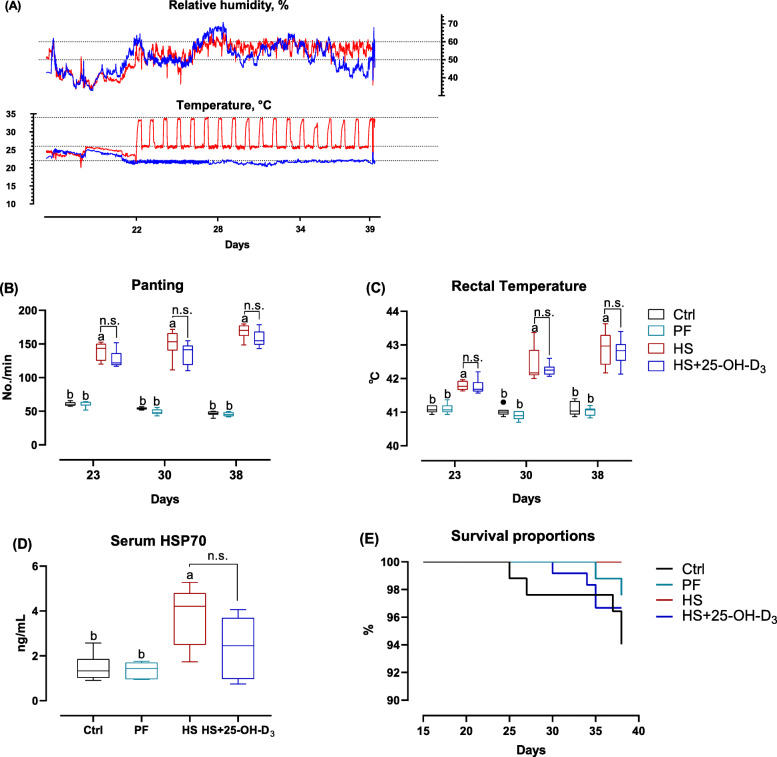
Temperature and relative humidity in thermo-neutral room (blue) and HS room (red) from 22 to 39 d (**A**), and effect of HS and supplemental 25-OH-D_3_ in broilers on **B** panting, **C** rectal temperature, **D** serum heat shock protein 70 (HSP70) on d 39 and **E** survival. Treatments Ctrl, PF and HS without common superscript are significantly different at *P* < 0.05. n.s., denotes no significant difference between HS and HS + 25-OH-D_3_ at *P* < 0.05

Animal procedures were approved by the Ethics Committee of the Faculty of Veterinary Medicine, Ghent University (Belgium) for the humane care and use of animals in research (approval No. 2019–87). Male chicks from the Ross 308 strain (Vervaeke-Belavi, Tielt, Belgium) were housed in floor pens (1.0 × 0.9 m) in a climate-controlled facility. The temperature in the room was 34 °C for the first week and then gradually lowered to 22 °C by 22 d based on normal management practices. The light schedule was 23 L:1D and 18 L:6D (18 L from 04:00 to 22:00) during 1–7 d and beyond, respectively. The broilers were vaccinated at 1 d of age against Newcastle Disease and Infectious Bronchitis at the hatchery facilities. At 18 d of age the vaccination against Newcastle Disease was repeated with Nobilis ND Clone 30 by spraying. At 15 d of age, birds with mean 0.48 ± 0.003 kg body weight (BW), were randomly into 4 treatment groups: (1) the control group (Ctrl; constant 22 °C from 22 d onward and ad libitum feeding the basal diets, *n* = 7), (2) the HS with ad libitum intake (from 22 d onward 34 °C for 7 h daily with RH between 50% and 60% and the rest of day at 26 °C, *n* = 9), (3) the pair-fed group (PF; constant 22 °C from 22 d onward and pair-fed to HS group), and (4) the 25-OH-D_3_ diet ad libitum under HS (HS + 25-OH-D_3_; *n* = 10); the 25-OH-D_3_ concentration was 0.069 mg/kg diet and provided by DSM Ltd. (DSM Nutritional Products, Basel, Switzerland). Each replicate consisted of 12 birds (density of 13.3 birds/m^2^) and number of replicates per treatment was established based on statistical power analysis. Dietary regimes were followed in grower and finisher phase (15–21 d) and finisher phase (22–39 d). Pair feeding of PF was established in finisher period by measuring daily FI of HS group, and providing this amount the next day in three meals across the light hours of the day. PF was included to decipher whether the effects of HS are solely due to reduced FI or not. The chronic cyclic HS was thus implemented in the finisher phase (22–39 d) as shown in Fig. 1A. Diets included starter (1–14 d), grower (15–21 d) and finisher (22–39 d) diets, and were supplied as pellets (Table S1). The dry matter (DM), crude protein (CP), ether extract, ash, Ca, and P of diets were analyzed as described elsewhere [36]. Vitamin D_3_ and 25-OH-D_3_ in diets were quantitated by DSM Nutritional Products (Basel, Switzerland). Specifically, after saponification with potassium hydroxide alkaline ethanol solution and extraction with cyclohexane solvent, vitamin D_3_ was quantified by a reversed-phase high pressure liquid chromatography with tandem mass spectrometry (HPLC-MSMS) using 6, 19, 19-trideuterocitamin D_3_ as stable isotope labelled internal standard. For 25-OH-D_3,_ after addition of the internal standard, the sample was saponified and 25-OH-D_3_ was extracted with methyl tert-butyl ether. Then, the extract was dried by evaporation and then analyzed with reversed phase HPLC-MSMS detection. The quantification was carried out by using d6–25-hydroxy cholecalciferol as internal standard. The results of dietary nutrient analysis confirmed proper preparation of experimental diets (Table S2).

### Sample collection and procedures

In the present study, BW, FI, and mortality by pen were recorded at 15, 22, and 39 d. BW gain and feed conversion as the feed to gain ratio (F: G) was calculated on a per-pen basis. Panting frequency and rectal temperature of three birds randomly taken from each pen were measured at d 23, 30 and 38 as described by Majdeddin et al. [37]. Two birds per pen with weight close to average weight of the pen were sampled on d 39 (4 h after starting high temperature for birds from the HS room). The 1st bird was used for sample collection after euthanasia using sodium pentobarbital (30 mg/kg of BW) injected intravenously via the brachial vein. Blood was taken by puncture in the heart with 80 mm needle 22 G and divided it into three parts, one part was immediately dropped into K_2_EDTA-tube used for fixation of blood by the addition of Transfix® reagent (Caltag Medsystems, Ltd., UK) for whole blood, another part was thrown into K_2_EDTA-anticoagulant tubes and then centrifuged at 3,000 × *g*/15 min at 4 °C to obtain plasma, and the last one was centrifuged at 4,000 × *g*/15 min at 4 °C for serum after complete coagulation, and subsequently mid-duodenal and mid-ileal (removing one centimeter right in the middle for histology analysis) mucosa, kidney, caecal contents, left tibia (the proximal end), and tibial marrow were collected, then snap frozen in liquid nitrogen and stored (− 80 °C) until analysis. Thymus, spleen, and bursa of Fabricius were excised and weighed, and calculated as relative weight of organ (g/100 g BW). Right tibia and mid-ileum were dissected and rapidly immersed in phosphate-buffered formaldehyde for histology analysis. The 2nd bird was also euthanized, and the left tibia was removed for micro computed tomography (Micro-CT) analysis. The right tibia was harvested, length, and width (at 50% of length) of tibia were measured after removal of soft tissues.

### Gait score

After d 39, birds remained subjected to their respective HS or thermoneutral condition, and on d 40, walking ability of 3 randomly selected birds per pen was scored on a 6-point scale from 0 (completely normal) to 5 (unable to stand) according to the method of Knowles et al. [38]. Each bird was encouraged to walk approximately 10 m. The use of a stick was gently employed if the bird showed hesitation or unwillingness. The evaluator sat on the floor at eye level viewing the back of the broiler’s legs and then scored.

### Gut permeability

Gut permeability was evaluated by determining the ability of fluorescein isothiocyanate dextran (FITC-d; 4 kDa, Sigma, Overijse, Belgium) to cross from the intestinal lumen into circulation [39]. On d 40, 33 birds (1 chicken per pen) close to the pen average weight were selected, and all chickens received orally FITC-d (4.16 mg/kg). Blood was collected at 1 h post FITC-d administration and the fluorescence of serum FITC-d was determined using a Thermo Fluoroskan Ascent FL fluorometer (Thermo Scientific, Merelbeke, Belgium) at excitation and emission wavelengths of 485 and 530 nm, respectively. The content of FITC-d was calculated from standard curves generated by the serial dilution of FITC-d.

### Blood hematology and biochemistry

Freshly drawn fixated whole bloods from 39 d were used to assess numbers of thrombocytes and white blood cell differentials as outlined by Seliger et al. [40]. This automated analysis of chicken blood is based on flow cytometry using an anti-CD45 monoclonal antibody in combination with selected subset specific markers. Serum heat shock protein (HSP70) was analyzed using ELISA kit (Chicken Heat Shock Protein 70, CSB-E11196Ch, Cusabio, Wuhan, China). Plasma 25-OH-D_3_ and 24(R), 25-(OH)_2_-D_3_ concentrations were determined by using reversed phase HPLC-MSMS detection (DSM Nutritional Products, Basel, Switzerland). Plasma P was determined by ammonium molybdate (Abbott Laboratories, Germany). Plasma Ca was determined by the chromogenic complex formed between Ca ions and o-cresol phthalein (MAK022; Sigma-Aldrich, Overijse, Belgium). As bone turnover markers, C-terminal cross-linked telopeptide of type I collagen (CTx) in plasma was measured using an immunoassay (Cobas; Roche Diagnostics) with a LOQ of 10 ng/L. Plasma alkaline phosphatase (ALP) was measured by catalyzing the hydrolysis of colorless *p*-nitrophenyl phosphate to give *p*-nitrophenol using commercially available assay kits (Abbott Laboratories, Germany).

### Mechanical testing of tibia

After the measurements of length and width, all right tibias were used for bone quality analysis. Biomechanical testing was performed by the 3-point bending method using a Mecmesin BFG 25000 N force gauge (Mecmesin, Slinfold, UK) at a constant 50 kg load cell. Each bone was loaded on its anterior aspect, at the mid-point between the bottom supports, at the precise mid-point along its length. Loading proceeded at a constant rate (12 mm/min) up to the breaking of the bone. Force-displacement data were collected to calculate whole bone stiffness (slope of the linear portion of the load-displacement curve), yield load, fracture load and area-under-the-curve (AUC). The yield point was defined as the load at which the load-deformation relationship ceased to be linear [41].

### Fat-free weight, density and ash content of tibia

After mechanical testing, the tibia of each bird was used for tibial fat-free weight (g) and density (g/cm^3^), which was defined as bone fat free weight (g) divided by tibial volume (cm^3^). In detail, after removing the bone marrow cavities liquid with absorbent paper, the tibia volume was measured based on the quantum of water overflowing from a fully filled container when the measured tibia was inserted into a 100 mL cylinder, Hereafter, these bone samples were air-dried for 24 h at room temperature, extracted by ethyl ether for 48 h, oven-dried at 108 °C for 24 h for dry defatted bone weight determination. Subsequently, dry-defatted tibia was ashed in a muffle furnace at 550 °C for 24 h and the ash was measured on the basis of the percentage of dry-defatted weight.

### Micro-CT

The left tibia was prepared for micro-CT imaging. To avoid an influence of the water content within the bone, soft tissue was removed from the tibia and then tibia was dried at 65 °C. Preliminary tests showed that the procedure did not affect the imaging process. Furthermore, it allowed for a more uniform positioning. The samples were imaged in the custom-designed micro-CT scanner HECTOR of the Ghent University Center for X-ray Tomography (UGCT; www.ugct.ugent.be) [42]. For each micro-CT scan, 2,001 projection images were acquired at an exposure time of 1,000 ms for each projection, covering the full 360° angular range. A tube voltage of 130 kV was employed at 10 W target power. An additional filter of 0.5 mm Al was used to reduce beam hardening effects. Using geometrical magnification, the reconstructed isotropic voxel size was 22^3^ μm^3^. Projection data were reconstructed to a stack of 2D slices using the in-house developed reconstruction platform Octopus Reconstruction [43]. Next, the micro-CT images were analyzed using custom-written scripts in Image J (National Institutes of Health, USA). The volume of interested cortical bone was chosen from the tibial mid-diaphysis and extended proximal-distally for a total length of 37 mm. The center of the diaphysis of the tibia was automatically classified into trabeculae, cortex and ‘total cortex’ (including pores), using an algorithm similar to that of Buie et al. [44]. The total volume and the volume of cortical bone and trabecular bone were quantified, and bone volume to total volume ratio (BV/TV), cortical thickness and trabecular thickness were calculated. To measure the trabecular bone in the proximal end of the tibia (metaphysis), a region 4 mm in length was determined, starting 9 mm below the surface of the condyles. To exclude denser cortical regions at the bone surface, the outer 0.5 mm of the bone surface was removed from the region of interest. The BV/TV of trabecular bone and trabecular thickness were calculated. The average thickness of the structures was measured using the thickness plugin from Bone J [45].

### Ileum and bone histological analysis

Formalin-fixed ileal samples were dehydrated, embedded, sliced into 5-μm transects, and stained with hematoxylin and eosin (H&E), and subsequently villus height (V) and crypt depth (C) of at least ten well-oriented villi, were measured and the ratio of villus height to crypt depth (V/C) was calculated [46]. In addition, to identify osteoclast count on the external surface of the bone, the fixed proximal tibia samples were decalcified in 14% EDTA (pH 7.4) for 21 d, embedded in paraffin, frontally sectioned into 10-μm slices, and subjected to tartrate-resistant acid phosphatase (TRAP) bone staining using the leukocyte acid phosphatase assay kit (Sigma-Aldrich, Overijse, Belgium) according to the instructions. All histomorphometry data acquisition was performed using an Olympus BX61 microscope and image analysis software (Olympus, Aartselaar, Belgium).

### Oxidative stress

The buffered aqueous extracts of ileal mucosae were prepared after mixing with 1% Triton X-100 phosphate buffer (pH 7; 50 mmol/L), and then homogenized, centrifuged, and filtrated. Subsequently, total malonaldehyde (MDA) content in buffered aqueous extracts and plasma were tested using the thiobarbituric acid reactive substances test according to the method of Grotto et al. [47] with slight modifications using spectrophotometry at 532 nm. The GSH-Px activity of buffered aqueous extracts and plasma, defined as the amount of sample (g) required to oxidize 1 μmol of 2, 4-dinitrophenylhydrazine (DNPH) per minute at 25 °C, were quantified based on the dynamical alteration in the oxidation of NADPH and reaction time using Multi-Mode Microplate Readers at 340 nm [48]. The SOD activity represented as percentage inhibition of autoxidation of pyrogallol was determined by observing the increase in absorbance at 420 nm for 5 min by spectrophotometry [49].

### Gene expression assays

Total RNA was extracted from the duodenal and ileal mucosa, kidney, tibia and marrow using the Trizol reagent (Sigma-Aldrich, Overijse, Belgium) and RNA concentrations were quantified using a spectrophotometer (Nanodrop 2000; Thermo Fisher Scientific Inc., Merelbeke, Belgium). First-strand complementary DNA (Cdna) was reverse-transcribed from 200 ng of total RNA using the PrimeScript™ RT Reagent Kit (RR037A, Takara; Saint-Germain-en-Laye, France). The obtained cDNA was used to determine the mRNA expression of genes of interest by ABI 7500 RT-PCR detection system (Applied Biosystems, Merelbeke, Belgium), using Fast SYBR Green Master Mix (Takara, Saint-Germain-en-Laye, France). Primers were designed using Primer 3 (Table S3). The standard curve method was used to estimate reaction efficiency (slope). Relative gene expression was quantified by normalizing to the expression of glyceraldehyde-3-phosphate dehydrogenase (*GAPDH*) and *β-actin* mRNA, with Ctrl equaling to around 1.

### Gut microbiome analysis

The microbial composition of caecal contents was evaluated by 16S ribosomal RNA (rRNA) profiling. Specifically, genomic DNA was extracted using PSP Spin Stool DNA Plus Kit (Invitek, Westburg, Netherlands) according to the manufacturer’s instructions. After evaluation of DNA concentration and purity, the V1–V3 hypervariable region of the bacterial 16S rRNA was amplified using the specific primer (Forward primer: 5′-GAGAGTTTGATYMTGGCTCAG-3′ and reverse primers: 5′-ACCGCGGCTGCTGGCAC-30). Based upon the purified PCR products, Illumina sequencing libraries were generated using V3 chemistry kit and sequenced on an Illumina Miseq platform (Illumina, San Diego, USA). The obtained sequences were processed using MOTHUR (software package v1.39.5) for alignment and clustering [50], while the VSEARCH algorithm was utilized for chimera detection [51]. The final reads were clustered as operational taxonomic units (OTUs) with a 97% similarity threshold. The OTUs were further assigned to species level using the BLASTN algorithm based on the SILVA database (v1.32) of full-length 16S rRNA sequences [52]. The alpha diversity was evaluated with MOTHUR at the genus level by calculating the Chao1 richness index (richness), reciprocal Simpson biodiversity index (diversity), and Simpson evenness index (evenness). Beta-diversity at genus level was estimated by calculating Bray-Curtis dissimilarity and visualized with principal co-ordinates analysis (PCoA). Sequences generated in the current study have been deposited in the NCBI database under the accession number PRJNA694510.

### Cecal short-chain fatty acid (SCFA) analysis

Cecal contents were weighted, and approximately 1 g was dissolved in 5.5 mL 10% formic acid containing 0.5 mg ethyl butyric acid as the internal standard. After filtration and centrifugion the supernatants were used to determine total SCFA, acetate, propionate, butyrate, iso-butyrate, valerate, and iso-valerate concentrations using a gas chromatography on a Shimadzu 2010 (Shimadzu Corporation, ‘s-Hertogenbosch, The Netherlands) equipped with a flame ionization detector. The conditions were used as described previously [53].

### Statistical analyses

The data obtained were analyzed by the Shapiro-Wilk and Levene’s test to assess normal distribution and homogeneity of variances. One-way ANOVA followed by Tukey’s test for multiple comparisons (normal distribution) or Kruskal-Wallis test followed by Dunn’s multiple comparisons (non-normal distribution) was performed to elucidate a potential response by HS among Ctrl, PF, and HS groups on performance and physiological indices. Differences between HS and HS + 25-OH-D_3_ groups were evaluated using a two-tailed unpaired *t*-test or the Mann-Whitney U test for normally or non-normally distributed datasets, respectively. For estimating mortality, survival curves were constructed using Kaplan-Meier method and statistically analyzed by the log-rank test for trend. Values are given as mean ± standard error. Differences in alpha diversity metrices were evaluated similarly. Permutational multivariate analysis of variance was applied to test the effect of treatment on overall community composition on Bray-Curtis distance. *P* < 0.05 was considered statistically significant. A trend was regarded as *P* < 0.1.

## Results

### Growth performance

As illustrated in Fig. 1, implementing the chronic cyclic HS to broilers from 22 to 39 d (Fig. 1A) increased the respiratory rate expressed as panting and rectal temperature at 23, 30, and 38 d of age (Fig. 1B, C; *P* < 0.05), as well as serum HSP70 concentration at 39 d (Fig. 1D; *P* < 0.01) as compared with Ctrl or PF birds. Dietary 25-OH-D_3_ supplementation did not affect panting, rectal temperature, serum HSP70 and survival in comparison to HS (Fig. 1B-E; *P* > 0.05).

Data of growth performance show that HS notably decreased BW at 39 d, BW gain and FI (22 to 39 d) (*P* < 0.05), but it did not affect F: G. HS effects on production were at the levels of PF. However, PF birds had higher F: G than Ctrl birds (from 22 to 39 d, *P* < 0.05). The birds supplemented with 25-OH-D_3_ had higher BW at 39 d and BW gain (22 to 39 d) than the birds fed without 25-OH-D_3_ during HS (Table 1; *P* < 0.05). Regarding mortality, neither HS or 25-OH-D_3_ changed mortality (*P* > 0.05), i.e. bead birds were 5/84 (birds died/total birds), 2/84, 0/108, and 4/120 in Ctrl, PF, HS, and HS + 25-OH-D_3_ group, respectively (Fig. 1E).

**Table 1:** Effect of HS and supplemental 25-hydroxycholecalciferol (25-OH-D_3_) on performances of broilers

Items	Ctrl	PF	HS	HS + 25-OH-D_3_
**BW, g**				
15 d	483.7 ± 0.53	483.7 ± 1.00	483.9 ± 0.80	483.1 ± 0.88
21 d	1,028.6 ± 22.96	1,027.1 ± 32.54	1,027.8 ± 13.82	1,024.3 ± 17.34
39 d	3,080.9 ± 45.66^a^	2,779.4 ± 33.65^b^	2,827.4 ± 55.83^b^	2,888.5 ± 57.91*
**Gain, g**				
15–21 d	544.9 ± 23.04	543.4 ± 32.61	543.9 ± 13.76	541.3 ± 15.33
22–39 d	2,052.3 ± 40.82^a^	1,752.3 ± 29.80^b^	1,799.6 ± 55.68^b^	1,864.1 ± 67.93*
15–39 d	2,518.2 ± 159.18^a^	2,276.1 ± 66.46^b^	2,343.5 ± 56.27^b^	2,373.7 ± 79.41
**FI, g**				
15–21 d	704.7 ± 14.94	713.8 ± 20.17	706.8 ± 9.39	709.8 ± 19.02
22–39 d	3,133.7 ± 39.93^a^	2,783.4 ± 55.54^b^	2,785.5 ± 39.25^b^	2,837.5 ± 68.95
15–39 d	3,711.3 ± 214.42^a^	3,464.6 ± 46.25^b^	3,490.6 ± 48.52^b^	3,498.6 ± 124.70
**F: G, g/g**				
15–21 d	1.29 ± 0.030	1.32 ± 0.046	1.30 ± 0.024	1.31 ± 0.043
22–39 d	1.53 ± 0.014^b^	1.59 ± 0.028^a^	1.55 ± 0.043^ab^	1.52 ± 0.033
15–39 d	1.47 ± 0.013^b^	1.52 ± 0.029^a^	1.49 ± 0.030^b^	1.47 ± 0.027

Data represent means with standard error;

Treatments Ctrl, PF and HS without common superscript are significantly different at *P* < 0.05. * denotes significant difference between HS and HS + 25-OH-D_3_ at *P* < 0.05;

*BW* Body weight, *FI* Feed intake, *F: G* Feed intake to gain ratio

### Gait score and bone characteristics

As shown in Table 2, HS did not result in difference in gait score, but dietary supplementation with 25-OH-D_3_ improved the walking ability of birds under HS (*P* < 0.05). The positive outcome for 25-OH-D_3_ was not associated with tibial growth, evidenced by similar tibia length, width, and fat-free weight. Mechanical testing analysis indicated a decrease in the mechanical properties in HS birds, i.e. lower fracture load in HS compared with Ctrl and PF birds (*P* < 0.05), which was restored by supplementation of 25-OH-D_3_ (*P* < 0.05). Also, HS did not affect tibia slope compared to Ctrl and PF groups, but slope was increased by supplementing 25-OH-D_3_ to heat-stressed birds (*P* < 0.01). Additionally, HS reduced both yield load and AUC to a level that was not significantly different from control birds, which was slightly prevented with dietary 25-OH-D_3_ treatment (both *P* > 0.05).

**Table 2:** Effect of HS and supplemental 25-hydroxycholecalciferol (25-OH-D_3_) on gait score (d 40) and tibia bone characteristics (d 39) of broilers

Items	Ctrl	PF	HS	HS + 25-OH-D_3_
**Gait score**	2.10 ± 0.28	1.38 ± 0.11	1.93 ± 0.23	1.37 ± 0.12*
**Bone growth**				
Length, mm	104.96 ± 1.80	103.26 ± 1.47	105.30 ± 1.11	105.31 ± 1.07
Width, mm	9.70 ± 0.23	9.96 ± 0.20	9.39 ± 0.21	9.50 ± 0.25
Fat-free weight, g	7.53 ± 0.25	7.51 ± 0.16	7.29 ± 0.18	7.29 ± 0.15
**Bone mineralization**				
Ash, %Fat-free weight	45.71 ± 0.26^a^	46.08 ± 0.41^a^	43.91 ± 0.59^b^	45.92 ± 0.42*
Density, g/mL	0.45 ± 0.01	0.44 ± 0.01	0.42 ± 0.01	0.45 ± 0.01*
Cortical thickness in diaphysis, μm	25.18 ± 1.80	26.06 ± 2.39	24.32 ± 0.96	28.25 ± 1.65*
BV/TV in diaphysis, %	0.56 ± 0.01	0.55 ± 0.01	0.54 ± 0.01	0.55 ± 0.01
Trabecular thickness in metaphysis, μm	4.14 ± 0.05	4.17 ± 0.09	4.19 ± 0.05	4.32 ± 0.03*
BV/TV in metaphysis, %	0.25 ± 0.01	0.27 ± 0.03	0.24 ± 0.01	0.25 ± 0.01
**Bone mechanical properties**				
Fracture load, N	708.33 ± 23.86^a^	725 ± 28.14^a^	587.5 ± 33.74^b^	707.14 ± 33.5*
Yield load, N	710.65 ± 77.63	678.56 ± 42.13	594.27 ± 51.06	769.00 ± 69.67
Slope, N/mm	310.91 ± 62.24	324.98 ± 82.38	296.30 ± 24.34	472.13 ± 50.13*
AUC, N × mm × 10^3^	3.36 ± 0.36	3.14 ± 0.39	2.63 ± 0.21	3.15 ± 0.34

Data represent means with standard error;

Treatments Ctrl, PF and HS without common superscript are significantly different at *P* < 0.05. * denotes significant difference between HS and HS + 25-OH-D_3_ at *P* < 0.05;

*AUC* Area under the load-displacement curve, *BV/TV* Bone volume/Total volume

Moreover, HS broilers exhibited decreased ash (*P* < 0.01) and density (*P* = 0.055) of tibia compared with Ctrl and PF birds, and birds fed 25-OH-D_3_ diets yielded increased mineral deposition of tibia evidenced by increased ash and density when compared with HS broilers (both *P* < 0.05). Further analysis with micro-CT showed that BV/TV and thickness of both trabecular and cortical bone in the diaphysis was not notably affected by HS as compared with Ctrl or PF broilers (*P* > 0.05). Under the HS condition, dietary 25-OH-D_3_ supplementation remarkably increased the cortical thickness in diaphysis and the trabecular thickness in metaphysis (*P* < 0.05), although it did not apparently change the BV/TV of both diaphysis and metaphysis.

### Ca, P, and vitamin D_3_ homeostasis

**Fig. 2:**
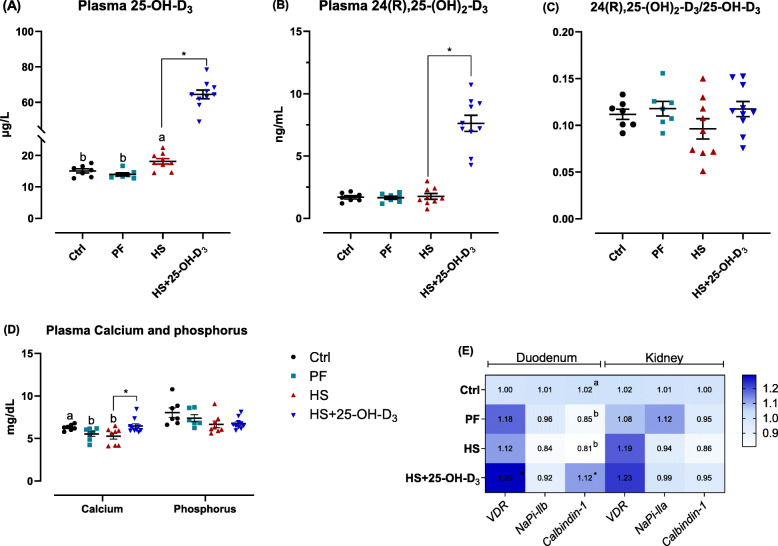
Effect of HS and supplemental 25-OH-D_3_ in broilers at d 39 on Ca and P homeostasis. Plasma **A** 25-OH-D_3_, and **B** 24(R), 25-dihydroxyvitamin D_3_ (24(R), 25-(OH)_2_-D_3_)_,_ **C** ratio 24(R), 25-(OH)_2_-D_3_ to 25-OH-D_3_, and **D** calcium and phosphorus. mRNA abundance of **E** RT-PCR analysis for mRNA expression of vitamin D receptor (*VDR*), sodium-dependent phosphorus transport protein II (*NaPi-IIb*) and *calbindin-1* in duodenum, as well as *VDR*, *NaPi-IIa* and *calbindin-1* in kidney. Treatments Ctrl, PF and HS without common superscript are significantly different at *P* < 0.05. * denotes significant difference between HS and HS + 25-OH-D_3_ at *P* < 0.05

Regarding the homeostasis of Ca, P, and vitamin D_3_, differences in plasma 25-OH-D_3_ concentration were found between Ctrl, PF, and HS groups (*P* < 0.01), but 24(R), 25-(OH)_2_-D_3_ and the ratio of 24(R), 25-(OH)_2_-D_3_ to 25-OH-D_3_ were similar among Ctrl, PF and HS groups (Fig. 2A-C). Dietary 25-OH-D_3_ addition markedly elevated plasma Ca, 25-OH-D_3_ and 24(R), 25-(OH)_2_-D_3_ levels (Fig. 2A-D; *P* < 0.05). In addition, compared with Ctrl broilers, birds from HS and PF groups displayed decreased Ca content (*P* < 0.05) and comparable levels of plasma P (*P* > 0.05) (Fig. 2D). No difference was observed among Ctrl, PF, and HS groups in terms of the mRNA expressions of vitamin D receptor (*VDR*) and sodium-dependent phosphorus transport protein IIb (*NaPi-IIb*) in the duodenum and kidney, neither of *calbindin-1* in the kidney (Fig. 2E; *P* > 0.05). However, heat exposure resulted in downregulated *calbindin-1* mRNA abundance in the duodenum when compared to Ctrl, but not when compared to PF groups (*P* < 0.05). Dietary 25-OH-D_3_ addition markedly increased mRNA expression of the duodenal *VDR* (trend, *P* = 0.052) and *calbindin-1* (Fig. 2E).

### Intestinal barrier

**Fig. 3:**
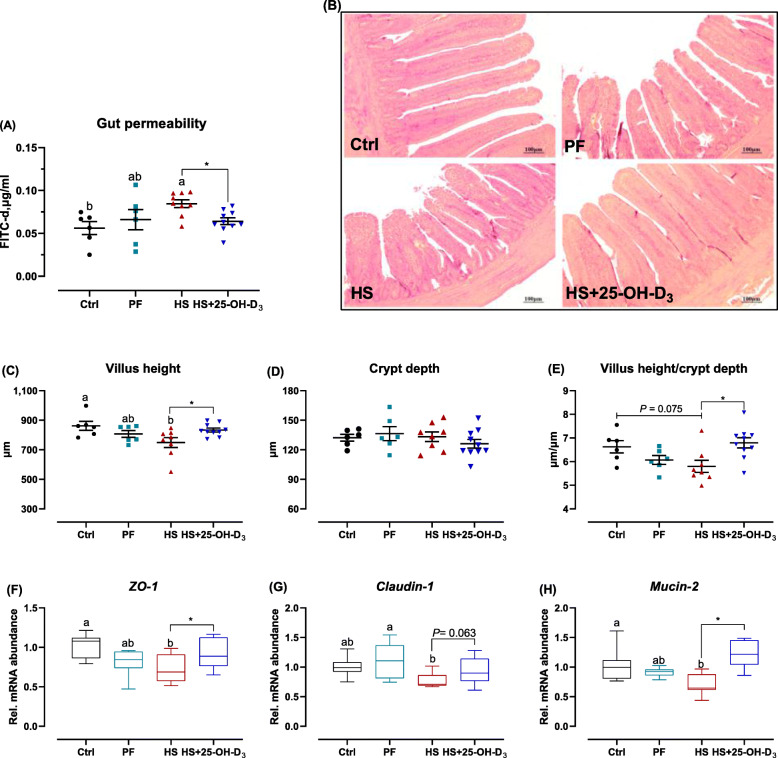
Effect of HS and supplemental 25-OH-D_3_ in broilers at d 39 on **A** gut permeability and intestinal barrier, **B** hematoxylin and eosin (H&E) staining (× 100), **C** villus height, **D** crypt depth, and **E** their ratio, mRNA abundance of **F** zonula occludens-1 (*ZO-1*), **G** *claudin-1* and **H** *mucin-2*. Treatments Ctrl, PF and HS without common superscript are significantly different at *P* < 0.05. * denotes significant difference between HS and HS + 25-OH-D_3_ at *P* < 0.05

Direct assessment of permeability using FITC-d suggests that HS significantly increased intestinal permeability, evidenced by higher serum FITC-d concentration (Fig. 3A; *P* < 0.05). Further examination of V and C confirmed that HS birds had a distinct decreased V (*P* < 0.05), but unaffected C, and a tendency (*P* = 0.075) to decrease V/C ratio relative to Ctrl broilers (Fig. 3B-E). Analysis of the tight junction proteins (TJPs) shows that the expression of *ZO-1* and *claudin-1* was decreased in HS compared with Ctrl or PF birds (Fig. 3F, G; *P* < 0.05), and mRNA of mucin-2 was downregulated (Fig. 3H; *P* < 0.05). 25-OH-D_3_ treatment prevented HS-induced increases in permeability, and decreases in V, V/C ratio, and TJPs and *mucin-2* transcription (Fig. 3).

### Caecal microbiome

**Fig. 4:**
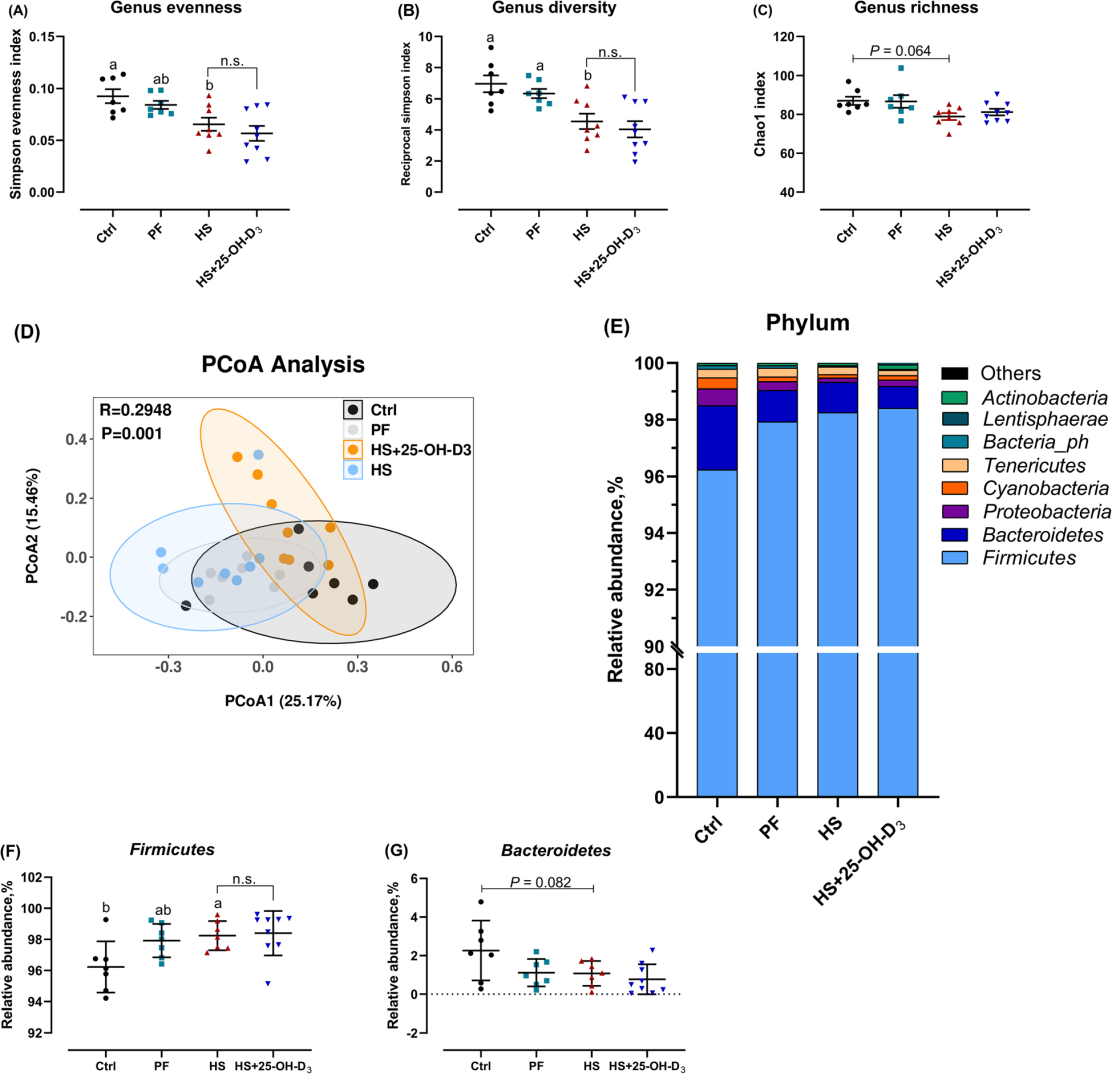
Effect of HS and supplemental 25-OH-D_3_ in broilers at d 39 on caecal microbiome; **A**-**C** Chao1 and Simpson indexes were used to assess diversity and evenness at genus level, **D** principle coordinate analysis plot (PCoA) of caecum microbiome diversity at species level based on Bray-Curtis dissimilarities; Ctrl vs. PF, *P* = 0.019; Ctrl. vs. HS, *P* = 0.006; PF vs. HS, *P* = 0.191; and HS vs. HS + 25-OH-D_3_, *P* = 0.002; R^2^ model = 0.33, **E** relative abundances of bacterial communities at phylum level, **F** Firmicutes, and **G** Bacteroidetes, **H**, **I** caecal short-chain fatty acid (SCFA). Treatments Ctrl, PF and HS without common superscript are significantly different at *P* < 0.05. n.s., denotes no significant difference between HS and HS + 25-OH-D_3_ at *P* < 0.05

Concerning the effects of HS and 25-OH-D_3_ on the caecal microbiome, the genus evenness (Simpson evenness index) and diversity (Simpson index) (both *P* < 0.05), but not richness (Chao1 index) (trend, *P* = 0.064) in the HS group were decreased compared with those in Ctrl and/or PF groups (Fig. 4A-C). According to the PCoA plot, the samples in the HS group did not form a distinct cluster from those in the PF group, but was separated from those in the Ctrl group (Fig. 4D). The compositions of caecal microbiota at phylum level differed among groups (Fig. 4E). The caecal microbiota in broilers was dominated by the Firmicutes and Bacteroidetes phyla. Specifically, Firmicutes levels were increased (*P* < 0.05) and Bacteroidetes levels (trend, *P* = 0.082) were decreased in HS birds compared with Ctrl birds and a trend for similar changes in PF birds was seen (Fig. 4F, G). However, no differences were found between HS and HS + 25-OH-D_3_ in either alpha diversity metrics or composition of the caecal microbiota at phylum level, though PCoA revealed a segregation between HS and HS + 25-OH-D_3_ (Fig. 4D; *P* > 0.05).

Total and individual SCFA contents of the caeca are also given in Table 3. HS and dietary 25-OH-D_3_ failed to result in changes in terms of the levels of total SCFA, acetate, iso-valerate and valerate (*P* > 0.05). However, heat exposure notably increased propionate, but decreased butyrate when compared with Ctrl group (both *P* < 0.05), resulting in higher level of propionate than butyrate for the HS group (*P* < 0.05). Unexpectedly, iso-butyrate was increased by restricted feeding (PF) that was similar with HS group as compared to Ctrl group.

**Table 3:** Effect of HS and supplemental 25-hydroxycholecalciferol (25-OH-D_3_) on short-chain fatty acid (SCFA, μmol/g) in caeca of broilers at d 39

Items	Ctrl	PF	HS	HS + 25-OH-D_3_
Total SCFA	117.03 ± 3.71	107.59 ± 2.75	115.48 ± 3.21	108.22 ± 4.63
Acetate	71.08 ± 1.17	70.54 ± 1.21	72.13 ± 0.67	71.31 ± 0.98
Propionate	9.77 ± 0.72^b^	11.76 ± 0.91^ab^	13.82 ± 1.05^a^	12.48 ± 0.88^n.s.^
Iso_ butyrate	0.69 ± 0.14^b^	1.38 ± 0.18^a^	0.99 ± 0.10^ab^	1.01 ± 0.06^n.s.^
Butyrate	16.38 ± 1.42^a^	13.34 ± 0.82^ab^	10.76 ± 0.67^b^	12.68 ± 1.05^n.s.^
Iso_ valerate	0.97 ± 0.19	1.74 ± 0.30	1.11 ± 0.12	1.21 ± 0.07
Valerate	1.11 ± 0.08	1.26 ± 0.09	1.19 ± 0.09	1.31 ± 0.04

Data represent means with standard error;

Treatments Ctrl, PF and HS without common superscript are significantly different at *P* < 0.05. n.s., denotes no significant difference between HS and HS + 25-OH-D_3_ at *P* < 0.05

### Oxidative stress

Heats stress and 25-(OH)-D_3_ affects oxidative stress as shown in Table 4. MDA concentration in the ileal mucosa was higher in birds subjected to HS as compared with Ctrl and PF birds (*P* < 0.05). Similarly, plasma MDA concentration was increased by HS as compared with PF birds, but not with Ctrl birds (*P* < 0.05). GSH-Px activity in ileal mucosa and plasma were not different among Ctrl, PF and HS (both *P* > 0.05), but SOD activity in ileal mucosa was decreased in HS compared with Ctrl or PF birds (*P* < 0.05). However, 25-OH-D_3_ treatment did not affect MDA content and antioxidant enzyme activities relative to HS broilers (*P* > 0.05).

**Table 4:** Effect of HS and supplemental 25-hydroxycholecalciferol (25-OH-D_3_) on indices of oxidative status of broilers at d 39

Items	Ctrl	PF	HS	HS + 25-OH-D_3_
**Ileum**				
MDA, nmol/g	15.57 ± 0.66	15.17 ± 0.96	17.8 ± 0.42	17.94 ± 0.25
GSH-Px, U/g	4.66 ± 0.51	4.75 ± 0.44	4.97 ± 0.25	4.55 ± 0.21
SOD, U/g	22.15 ± 0.38	20.28 ± 1.28	13.64 ± 0.40	13.82 ± 1.53
**Plasma**				
MDA, nmol/mL	16.25 ± 0.52	13.98 ± 0.75	19.45 ± 0.94	20.38 ± 0.68
GSH-Px, U/mL	1.22 ± 0.09	1.16 ± 0.08	1.13 ± 0.06	1.08 ± 0.07

Data represent means with standard error;

Treatments Ctrl, PF and HS without common superscript are significantly different at *P* < 0.05. n.s., denotes no significant difference between HS and HS + 25-OH-D_3_ at *P* < 0.05;

*MDA* Malondialdehyde, *GSH-Px* Glutathione peroxidase, *SOD* Superoxide dismutase

### Immune status in ileum and bone marrow

**Fig. 5:**
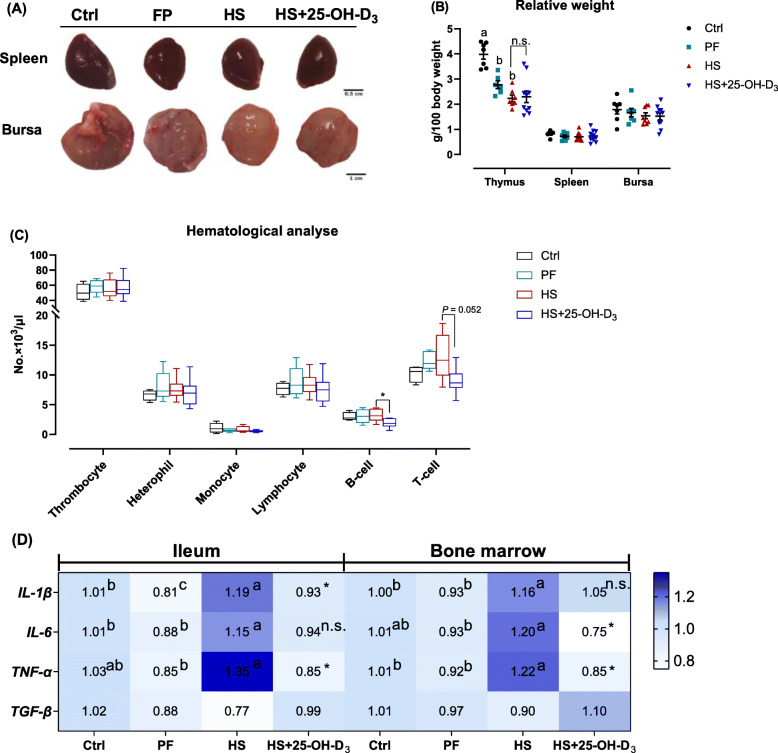
Effect of HS and supplemental 25-OH-D_3_ in broilers at d 39 on blood and immunological indices; **A** representative images of spleen and bursa, **B** relative weight of thymus, spleen and bursa, **C** detection of leukocytes and thrombocytes in whole blood, RT-PCR analysis of **D** pro-inflammatory cytokines (including interleukin (IL)-1β, IL-6 and tumor necrosis factor-α (TNF-α)) and anti-inflammatory cytokines transforming growth factor-β (TGF-β) in ileum and bone marrow, respectively. Treatments Ctrl, PF and HS without common superscript are significantly different at *P* < 0.05. n.s., denotes no significant difference between HS and HS + 25-OH-D_3_ at *P* < 0.05

In this study, HS reduced the relative weight of the thymus (*P* < 0.05), but it did not affect the relative weight of both the spleen and bursa (Fig. 5A, B). Hematological analysis of blood showed similar numbers of thrombocytes, heterophils, monocytes, lymphocytes and B-cells in Ctrl, PF and HS birds. A higher abundance of T-cells in HS birds as compared with Ctrl broilers was noticed but this did not reach significance (Fig. 5C; *P* > 0.05). As showed in Fig. 5D, the outcome of inflammation analysis revealed that the mRNA levels of proinflammatory factors, including *IL-1β*, *IL-6*, and *TNF-α*, were increased in the ileum of HS birds compared with Ctrl and/or PF broilers (*P* < 0.05). Transforming growth factor beta (*TGF-β*) mRNA expression, representing an anti-inflammatory response, showed a trend to decrease in HS broilers (*P* = 0.087). In bone marrow, HS birds exhibited an increased expression of *IL-1β*, *IL-6*, and *TNF-α* (*P* < 0.05), but similar *TGF-β* mRNA level in bone marrow when compared to Ctrl or PF birds.

Furthermore, birds fed 25-OH-D_3_ diets displayed a substantial decrease in B-cells (*P* < 0.05) and T-cells (*P* = 0.052) compared to HS broilers (Fig. 5C). Abundances of pro-inflammatory factors were correspondingly modulated by dietary 25-OH-D_3_ treatment, i.e. *IL-1β* and *TNF-α* expression in ileum were remarkably suppressed by 25-OH-D_3_ supplementation while a trend for higher mRNA abundance of *TGF-β* was seen (*P* = 0.078) (Fig. 5D). Similarly, the expressions of pro-inflammatory factors *IL-6* and *TNF-α* were also decreased in bone marrow of HS + 25-OH-D_3_ compared with HS birds (Fig. 5D; *P* < 0.05). However, the relative weight of immune organs and *TGF-β* mRNA expression in bone marrow did not change by 25-OH-D_3_ supplementation during HS (Fig. 5; *P* > 0.05).

### Bone resorption and formation

**Fig. 6:**
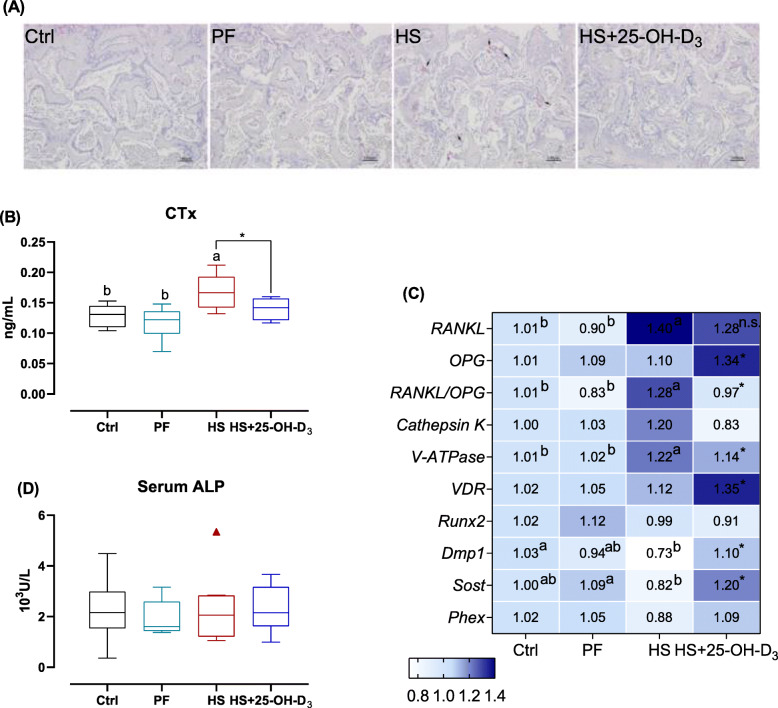
Effect of HS and supplemental 25-OH-D_3_ in broilers at d 39 on markers of bone metabolism. **A** Tartrate-resistant acid phosphatase (TRAP) staining of tibia sections. **B** Serum bone resorption biomarker C-terminal cross-linked telopeptide of type I collagen (CTx) concentrations were determined. **C** mRNA expression of bone remolding in the tibia proximal end were quantified. **D** Serum bone formation biomarker alkaline phosphatase activity (ALP) were analyzed. Treatments Ctrl, PF and HS without common superscript are significantly different at *P* < 0.05. n.s., denotes no significant difference between HS and HS + 25-OH-D_3_ at *P* < 0.05. RANKL: Receptor activator of nuclear Factor-κ B ligand; OPG: Osteoprotegerin; V-ATPase: Vacuolar-type H^+^-ATPase; VDR: Vitamin D receptor; Runx2: Runt-related transcription factor 2; Dmp1: Dentin matrix protein1; Phex: Phosphate regulating endopeptidase homolog x-linked

Heat exposure and dietary 25-OH-D_3_ administration significantly affected the bone resorption. Figure 6A shows that the circulatory level of CTx, that reflects bone resorption, was increased in HS compared with Ctrl or PF birds (*P* < 0.01), and then was depressed by dietary 25-OH-D_3_ treatment (*P* < 0.05). As shown in Fig. 6B, TRAP-positive cells from HS birds were also distinctly increased, and supplementation of 25-OH-D_3_ reduced the number of TRAP-positive cells in bone sections. As far as osteoclastogenesis-related factors in bone, HS increased the expression level of *RANKL* (*P* < 0.01) and did not change osteoprotegerin (*OPG*) mRNA abundance, and thus notably increased *RANKL*-to-*OPG* ratio when compared to Ctrl or PF groups (*P* < 0.05). Dietary 25-OH-D_3_ treatment elevated the expression level of *VDR*, and notably increased *OPG* mRNA expression, and consequently decreased *RANKL*-to-*OPG* ratio relative to HS broilers (Fig. 6C; *P* < 0.05). In addition, HS birds presented a significant increase mRNA expression level of *cathepsin K* than those from Ctrl group (*P* < 0.05), and subsequently prominent decreased *cathepsin K* mRNA level was also observed following dietary 25-OH-D_3_ manipulation under HS (Fig. 6C; *P* < 0.05). HS exposure or 25-OH-D_3_ supplementation did not affect vacuolar H^+^-ATPases (V-ATPase) transcription (Fig. 6C).

No obvious differences in the serum ALP activity, an indicator of bone formation, and runt related transcription factor 2 (*Runx2*) mRNA level reflecting osteogenesis were observed (Fig. 6C, D; both *P* > 0.05). Furthermore, with regard to the osteocyte-specific marker genes expression and when compared to Ctrl group, HS reduced the mRNA abundances of dentin matrix protein 1 (*Dmp1*) (*P* < 0.05), but the expression of sclerostin (*Sost*) and phosphate regulating endopeptidase homolog x-linked (*Phex*) appears to be similar between Ctrl and HS (Fig. 6C). Next, the birds consuming the 25-OH-D_3_ diet displayed a significant increase in *Sost, Dmp1,* and *Phex* (*P* = 0.090) relative to HS broilers.

## Discussion

Thermal environment has drawn greater attention to poultry producers because of its detrimental impacts, especially for meat-type poultry [1]. Exposure of birds to high ambient temperatures has shown to impair gut integrity and alter physiological homeostasis, such as systemic immune dysregulation, endocrine and bone disorders, microbiota perturbation, which result in poor performance [2, 3, 54]. Similarly, the deleterious effects of HS were confirmed in the current study by decreased BW, FI and higher F: G. To accommodate heat dissipation, broilers can enhance their respiratory rate in the high temperature environment [55], which is illustrated by the higher panting frequency in our study. Moreover, rectal temperature and serum HSP70, both indicators of HS, were elevated by heat exposure. Altogether, this indicates that the chronic cyclic HS model was successfully established in this study. In our study we also included a pair feeding treatment (PF), i.e. birds were housed under thermoneutral conditions and fed restrictively in the finisher period following FI of HS birds. Thereby, the appropriate daily amount of feed was provided in three meals across the light hours of the day. PF was included to decipher whether HS effects are solely due to reduced FI. Indeed, FI of PF was equal to HS, and this corresponded to approximately 89% of ad libitum (Ctrl). For some indices, they were similar between Ctrl and PF, and both were different from HS, which suggests that HS effects are not associated with reduced FI; or vice versa, Ctrl distinctly differing from both HS and PF might imply that HS is deteriorating bird physiology in part due to reduced FI. Finally, for some variables, PF is simply intermediate, not different from Ctrl or HS while HS is unlike Ctrl. It is plausible that precise interpretation depends on the variable considered, thus not straightforward. However, we observed during feeding times of PF birds that severe aggression occurred, and feed consumption may have been largely variable for individual birds. Moreover, consuming three extensive meals in short time versus multiple smaller meals ad libitum affects bird metabolism, and this might have confounded outcomes. Hereby, it is concluded that comparisons including PF should be interpreted cautiously.

Previous studies support a malignant role for HS in the regulation of bone health for birds. For example, imposing HS induced lower bone mass in broilers [2, 3], laying hens [4], and turkeys [5]. Negative effects of HS on tibia properties were also observed in this study. At the age of 39 d, results of whole bone mechanical testing of tibia demonstrate that the bones from HS-birds were mechanically inferior in most variables tested, especially fracture load. In addition, AUC is a measure of the amount of energy required to cause bone fracture, and ductile bones require a larger amount of energy to failure than brittle bones [41]. In our study, the AUC of tibia from the Ctrl and PF group was greater than that of tibia from the HS group in spite of not reaching statistical significance, indicating that imposing HS impaired tibial strength and mechanical properties of broilers. Similar to the results obtained in the current experiment, previous studies on tibia from 42-d-old broilers have shown that HS notably reduced breaking strength compared with the group reared in thermo-neutral temperatures [56]. Increased ambient temperature and stocking density were also found to decrease the maximum elastic strength and ultimate strength of tibia in 126-d-old turkeys [5]. Bruno et al. reported that heat exposure in broilers strikingly reduced tibial length and width [57], while investigations of Jankowski et al. in turkeys showed that both tibial weight and length were depressed by increased environmental temperature [5]. The results of the present study consistently indicate that tibia from HS birds had slightly lower bone weight, length, and width than those of the Ctrl group. Furthermore, supplementation with 25-OH-D_3_ was found to effectively increase the mechanical properties of tibia in birds subjected to HS, and to some extent, alleviate the damage to gait score during HS.

Such different mechanical properties can be attributed to architectural features of the bones, to material composition and bone mass, or to a combination of these [58]. In the present study, unfavorable effects of HS on tibial mineral composition were visible when evaluating ash and density. In the HS group, both tibia ash and density were decreased compared with the Ctrl and PF group, indicating poorer tibial mass in birds exposed to HS. The negative effects of high ambient temperature on the tibia in broilers observed in this study are in accordance with some previous reports, in which heat exposure caused a meaningful reduction in the content of ash, Ca, and P in broilers [2] and quails [59]. It should also be noted that 25-OH-D_3_ treatment caused positive changes in tibial mass as evidenced by apparent elevated bone ash, density, as well as the thickness of both trabecular and cortical bone, demonstrating that dietary supplementation with 25-OH-D_3_ can restore tibial mass induced by HS. Consistent with our findings, Sahin et al. showed that quails subjected to HS exhibited a significant decrease in tibia bone mineral density and tibial ash than those at thermoneutral environment, and these decreased bone parameters were linearly improved by 25-OH-D_3_ supplementation under HS [60]. These findings are in accordance with previous studies conducted in broilers [61, 62] and meat ducks [63].

To understand the mechanisms linking changes by HS and 25-OH-D_3_ to bone quality, it is useful to consider the three potential mechanisms: regulation of nutrient absorption [8], regulation of the intestinal barrier and immune system, and direct action on bone remodeling [9, 15]. Recent studies have shown that HS in broilers [64] and high-yielding dairy cows [65] caused an apparent reduction in absorption of Ca and P, along with the fact that HS compromises feed consumption. In the present study, the content of plasma Ca from the PF and HS groups were similar, both lower than Ctrl group, while a marginally lower P level was found in the plasma from the HS group, indicating that HS decreased Ca absorption and it was mainly associated with reduced food consumption. A compensatory increase in plasma 25-OH-D_3_ and a downregulated expression of *calbindin-1* mRNA in duodenum but not kidney further illustrates that lower plasma Ca mainly resulted from decreased Ca absorption in duodenum and not from decreased reabsorption in kidney. Data on 25-OH-D_3_ and 24(R), 25-(OH)_2_-D_3_ can also be used to evaluate the status of vitamin D from the perspective of the biological activity. 24(R), 25-(OH)_2_-D_3_ is the first catabolite of 25-OH-D_3_ arising from its hydroxylation by cytochrome P24A1 and the ratio of 24(R), 25-(OH)_2_-D_3_ to 25-OH-D_3_ has been served as an index of vitamin D_3_ clearance [66]. In the present study, dietary 25-OH-D_3_ treatment heightened plasma 25-OH-D_3_ and 24(R), 25-(OH)_2_-D_3_ concentrations but not pronouncedly changed the ratio of plasma 24(R), 25-(OH)_2_-D_3_ to 25-OH-D_3_ indicating that catabolism of vitamin D_3_ was not induced response to dietary 25-OH-D_3_ supplementation. Moreover, dietary 25-OH-D_3_ treatment markedly heightened plasma 25-OH-D_3_ concentration, which in turn has elevated plasma Ca level by inducing the mRNA expression of *calbindin-1* in duodenum of stressed-broilers. It is interesting to find out that supplemental 25-OH-D_3_ did not change plasma P level and the mRNA level of *NaP-IIb* and *NaP-IIa*, both responsible for regulating P transport, in duodenum and kidney, respectively [67]. Comparable findings were reported in broilers fed 2.5–5 μg/kg 1α-OH-D_3_. No significant differences in plasma P content and mRNA level of *NaP-IIb* and *NaP-IIa* were observed as compared with the birds fed control diets [68]. In addition, studies have also noticed that HS-induced morphological changes to the small intestine of broilers mainly consist of a shortened V, deepened C, and consequently reduced V/C [69]. Inevitably, this will lead to substantial reduction of intestinal digestion and absorption surface. This is consistent with our present data on the ileum histomorphology of birds. Furthermore, the increases in V and V/C by supplementation with 25-OH-D_3_ reflects enhanced digestion and absorption capacities of stressed-broilers [70]. Collectively, these results indicate that promoting Ca absorption and increasing absorption capacities of the small intestine through providing 25-OH-D_3_ can be crucial in alleviating the damage to bone mass due to HS-induced Ca deficiencies.

Impairment of gut integrity and regulation of systemic immune function might be another likely explanation for the differences in bone quality in the current study. It has been reported that HS directly impaired gut integrity and induced the activation of the innate immune system and systemic inflammation in bovine [10], rats [11], and laying hens [12]. The TJPs serves as the innate defense barrier, formed by ZO, claudins, occludin, and adherence junctions, along with the mucus that covers its surface. These protect the host against paracellular bacterial infiltration and penetration of toxic substrates [71]. Our results indicate lower *ZO-1*, *claudin-1*, and *mucin-2* mRNA abundance in HS birds in line with findings in bovines [10] and broilers [72]. Subsequently this resulted in intestinal leakage, instructed by higher serum FITC-d level. These alterations of intestinal permeability can be related to bone health, supported by the fact that increased intestinal permeability observed in diseases, such as inflammatory bowel disease, is correlated with bone loss [73]. The high-molecular-weight polymer MDY is known to protect intestinal epithelial integrity against injury. A previous study pointed out that chickens infected with *Salmonella* benefit from MDY and do not loose trabecular bone as compared to untreated birds [74]. The administration of vitamin D_3_ was able to abolish the effects of LPS by restoring the expression of ZO-1 and claudin-2 [31], and had a protective effect against pepsin-trypsin-resistant gliadin induced tight junction injuries both *in vitro* and *in vivo* [32]. This underscores the importance of dietary 25-OH-D_3_ supplementation on the gut-bone signaling axis.

More important, in the current study, HS caused significant changes in microbial composition as evidenced by decreased genus evenness and diversity, as well as forming a distinct cluster that was separated from Ctrl. Relative abundance at the phylum levels manifested in substantial increases in Firmicutes, and an apparent decrease in Bacteroidetes, normally both predominant phyla in the caecum of broilers and laying hens. The results are consistent with previous reports in broilers [13, 14], but inconsistent with some earlier studies saying that HS had no effect on the phyla composition in the caecum of laying hens [75]. The reasons for this change in relative abundance at phylum level by HS is not clear but may be depend on animal species, feed, location and environment, as suggested by Stanley et al. [76]. A decrease in Bacteroidetes abundance has been associated with intestinal inflammation such as IBD [77]. Interestingly, reduced Firmicutes: Bacteroidetes ratio was linked with lower inflammatory state and declined bone loss of mice [78]. Moreover, several reports highlighted the immunomodulatory capacities of SCFA and provided a direct mechanistic link between the gut microbiota and bone [79]. Accordingly, recent studies suggested that direct treatment of mice with SCFA or feeding with a high-fiber diet was characterized by an increase in bone mass and prevented postmenopausal and inflammation-induced bone loss, along with increased serum SCFA levels and inhibition of osteoclast differentiation and bone resorption [80], indicating SCFA functioning as promoter of bone health. A recent review says that the fluctuation in bone properties were associated with alterations in SCFA levels [15]. In our study, total SCFA concentrations in the caecal content were comparable among Ctrl, PF and HS. However, and rather astonishingly, propionate production was favored over butyrate production when birds were heat-stressed, which was contrary to thermo-neutral raised birds and to what is commonly understood from caecal fermentations in broilers. Yan et al. deems that a direct effect of SCFA on bone resorption *in vivo* is unlikely as the circulating concentration of these molecules is lower than the concentration needed to affect osteoclast differentiation [81]. 25-OH-D_3_ supplementation did not affect either Firmicutes or Bacteroidetes abundance in the current study. This is in contrast to previous studies deemed that vitamin D could modify the relative abundance of bacteria by stimulating the release of antimicrobial peptides from macrophages [33]. Thus, whether dietary 25-OH-D_3_ addition is critical for the effects of gut microbiota on bone mass remains elusive, and more subtle effects may occur.

It is well-established that some factors such as HS that induce microbiota dysbiosis and disturbs intestinal barrier skew the gut towards a more pro-inflammatory state [10, 74], which is often characterized by increased levels of pro-inflammatory factors such as IL-6 and TNF-α. In this study, using the ileum pro-inflammatory (IL-1β, IL-6 and TNF-α) and anti-inflammatory (TGF-β) factors as a marker of the balance between a pro- versus anti- inflammatory state, manifested that HS increased intestinal inflammation. Under conditions of impaired gut integrity, bacteria and their factors can translocate across the intestinal barrier to induce systemic inflammatory responses [73]. Heat-stressed quails exhibited higher serum TNF-α and C-reactive protein content [59]. Moreover, reduced relative weights of immune organs have been noticed in broilers under HS [2], which is confirmed by the results of the present study. It is well-established that inflammatory cytokines seem to have a direct role on bone remodeling via influencing the recruitment, maturation, proliferation, and activation of osteoclasts [82]. In this regard, rheumatoid arthritis proved to be a case for illustrating the relationship between inflammatory cytokines and bone metabolism. This autoimmune disease is characterized by inflammation with an increased expression of inflammatory cytokines such as TNF-α, leading to severe bone destruction mediated by osteoclasts [83]. TNF-α, which is secreted along with IL-1 from mononuclear cells, promotes osteoclastogenesis indirectly by stimulating RANKL expression, a facilitator of osteoclast differentiation, and enhancing RANKL binding to osteoclast precursors [84]. Mice with TNF-α induced arthritis were found to have increased circulation of osteoclast precursors. This was reversed by anti-TNF-α therapy and correlated with systemically increased TNF-α concentrations [85]. In addition, IL-6 is also another crucial inflammatory cytokine to stimulate osteoclast formation and function in vitro. IL-6 deficient mice were protected from a significant loss of bone mass together with an increase in bone turnover rates caused by estrogen depletion [86]. In line with these data, we found increased expression of *IL-1β*, *IL-6* and *TNF-α* in bone marrow of heat-stressed birds. Furthermore, the increased serum CTx content underscored that the bone resorption was enhanced in birds suffering from HS. Likely, this is a consequence of the increase in the number and activity of osteoclasts, i.e. apparent increases in TRAP-positive cells in the trabecular bone accompanied by upregulated mRNA level of *cathepsin K*, an enzyme secreted by mature osteoclasts to dissolve the organic components of bone in the process of bone resorption [87]. Specifically, RANKL binds to RANK that is expressed on the surface of osteoclast to induce osteoclast differentiation, whereas OPG acts as a decoy receptor by blocking the interaction of RANKL with its functional receptor RANK [88]. The higher *RANKL*-to-*OPG* ratio in HS birds than those in the Ctrl or PF broilers emphasizes the action of HS as inducer of osteoclast proliferation and differentiation. Furthermore, it is well known that B- and T-cells can foster profound changes in bone remodeling, despite the fact that the exact mechanisms are not well understood [19, 89]. Studies have shown that germ-free mice have higher bone mass with reduced number of CD4^+^ T cells in bone marrow associated with a decreased expression of TNF-α. This suggests that the gut microbiota is capable of shaping systemic immunity, and lower CD4^+^ T cells abundance is beneficial for bone mass [19]. However, disruption of the gut microbiota with antibiotics reduced CD20^+^ B and CD3^+^ T cell populations which was correlated with reduced whole-bone strength [89]. These inconsistent findings may be attributed to differences in animal age, sex, antibiotic usage, and genotype. Vitamin D showed anti-inflammatory functions [28]. Here, it decreased the number of B- and T-cells in blood, as well as depressed the expression of inflammatory factors, especially TNF-α, in both ileal mucosa and bone marrow of broilers upon HS. It was also found that birds fed 25-OH-D_3_ under HS condition displayed a decreased frequency of TRAP-positive cells, serum CTx content, and *cathepsin K* mRNA level. These data infer that 25-OH-D_3_ probably reduced bone resorption under HS by inhibiting the release of inflammatory factors that can promote osteoclastogenesis as a result of impaired intestinal integrity and/or dysbiosis. The final outcome is notably improved tibial mass. As a note, the current study did not include assessment of glucocorticoids, which usually is increased in birds under HS conditions [20], and can influence bone metabolism, leading to bone loss. Apart from reduction in bone formation through decreasing maturation and increasing apoptosis of osteoblasts, glucocorticoids may increase osteoclast maturation and activity, further contributing to increased bone resorption [20]. We therefore cannot rule out the possibility that the damage of tibia quality is not in part the result of glucocorticoids under HS.

Related to vitamin D, we further cannot ignore the possibility that vitamin D directly targets bone related cells governing bone formation and resorption processes [26, 27]. Our data clearly show that dietary supplementation with 25-OH-D_3_ markedly upregulated *VDR* mRNA abundance in tibia. Therefore, it is possible that increased 25-OH-D_3_ binding VDR located at tibia promotes the production of OPG by osteoblasts which abolishes osteoclastogenesis and inhibits matured osteoclast’s activity, evidenced by decreased *cathepsin K* transcription, and consequently decreases in bone resorption. Such metabolic effects of 25-OH-D_3_ are in line with a previous report that vitamin D decreased osteoclast numbers by reducing the expression of OPG and decreasing the RANKL-to-OPG ratio in mice [90]. Additional analyses of bone formation markers indicate that it was regulated by 25-OH-D_3_. To be specific, early osteoblast development was unaffected by dietary 25-OH-D_3_ under HS. This is evidenced by the unchanged serum ALP concentration and *Runx2* mRNA levels. However, an enhanced differentiated phenotype of bone cells was observed in birds fed 25-OH-D_3_ diets (upregulated *Dmp1*, *Phex* and *Sost* mRNA levels), which suggests the importance of 25-OH-D_3_ for the transition of osteoblasts to osteocytes. Although the anti-rachitic activity of vitamin D in vivo is well established, the impact of vitamin D on osteoblast differentiation is inconsistent. Some studies showed a stimulation of osteoblast differentiation and mineralization after vitamin D treatment [91, 92], but also an inhibition of osteoblast differentiation in mice was seen [90]. This discrepancy could be partly explained by the stage differentiation of osteoblast, Ca availability, or the dose of vitamin D used in these studies [92, 93]. Past studies examining vitamin D/VDR signaling and osteoblast differentiation also found that that the early stages of osteoblast differentiation and mineral deposition are independent of the vitamin D/VDR system, but it is particularly important during the late stages of osteoblast to osteocyte transition [94]. Thus, these results imply that the positive effect of dietary 25-OH-D_3_ on bone mass of birds suffering from HS is likely mediated by suppressing bone resorption but not by stimulating bone formation.

## Conclusion

**Fig. 7:**
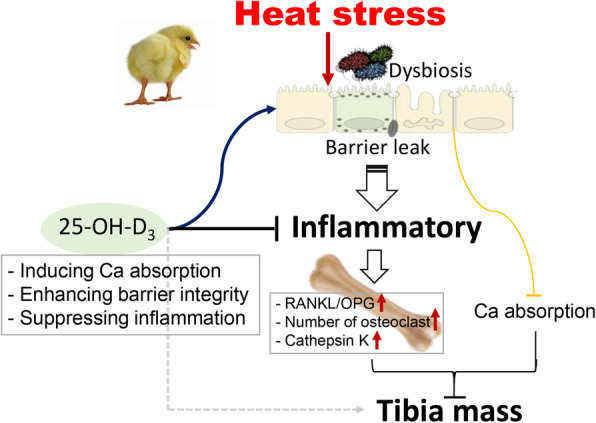
Proposed mechanism for how HS and 25-OH-D_3_ regulate bone mass. HS leads to decreased bone mass caused by reduced Ca absorption, disturbed microbiota, increased systemic inflammation, and induces bone resorption evidenced by the increased frequency and activity of osteoclast in bone marrow. Furthermore, dietary 25-OH-D_3_ treatment promotes Ca absorption, enhances intestinal integrity, and exertes ant-inflammatory properties, and consequently reverses tibia quality deterioration induced by HS

In this study, it was confirmed that HS has detrimental effects on bone health. We highlighted the importance of microbiota composition and intestinal barrier function in regulating bone quality. Disruption of intestinal integrity and dysbiosis by long-term exposure to high temperatures led to increased inflammation and bone resorption, and consequently impaired tibial quality. Furthermore, it was demonstrated that dietary supplementation with 25-OH-D_3_ was crucial for restoring bone health. Intestinal Ca absorption was enhanced and bone resorption reduced. These observations were associated with lower mRNA expression of inflammatory factors, and/or with direct modulation of osteoclast activity and maturation. Bone mass and strength was ameliorated by 25-OH-D_3_ when broilers were heat-stressed (Fig. 7).

## Supplementary Information

**Additional file 1: Table S1.** Composition and calculated nutrient content (as-fed). **Table S2.** Analysis of diet composition (as-fed). **Table S3.** The primers for quantitative real-time PCR.

## Abbreviations

25-OH-D_3_: 25-hydroxycholecalciferol
ALP: Alkaline phosphatase
GSH-Px: Glutathione peroxidase
HPLC-MSMS: Reversed-phase high pressure liquid chromatography with tandem mass spectrometry
IL: Interleukin
micro-CT: Micro computed tomography
RANKL: Receptor activator of nuclear factor-κ B ligand
SOD: Superoxide dismutase
TNF-α: Tumor necrosis factor alpha
TRAP: Tartrate-resistant acid phosphatase
ZO-1: Zonula occludens 1
