# Supplementary material for: 25-hydroxycholecalciferol reverses heat induced alterations in bone quality in finisher broilers associated with effects on intestinal integrity and inflammation
Source: J Anim Sci Biotechnol. 2021 Oct 8;12:104. doi: 10.1186/s40104-021-00627-6 (PMC8499578; doi:10.1186/s40104-021-00627-6)
Supplement: Supplementary file 1 — Additional file 1: Table S1. Composition and calculated nutrient content (as-fed). Table S2. Analysis of diet composition (as-fed). Table S3. The primers for quantitative real-time PCR. [file 40104_2021_627_MOESM1_ESM.docx]

**Table S1.** Composition and calculated nutrient content (as-fed)

| Item | Starter  (1-14 days) | Grower  (15-21 days) | Finisher  (22-39 days) |
| --- | --- | --- | --- |
| **Ingredients, %** | | | |
| Corn | 55.83 | 59.75 | 63.49 |
| Soybean meal | 26.43 | 24.22 | 22.28 |
| Toasted soybeans | 12.00 | 10.00 | 8.00 |
| Animal fat | 0.000 | 0.697 | 1.443 |
| Soybean oil | 0.967 | 1.000 | 1.000 |
| Monocalcium phosphate | 1.167 | 0.962 | 0.901 |
| Limestone | 1.451 | 1.352 | 1.213 |
| Sodium chloride | 0.237 | 0.248 | 0.262 |
| Sodium bicarbonate | 0.341 | 0.352 | 0.182 |
| Premix * | 0.500 | 0.500 | 0.500 |
| Choline chl. | 0.100 | 0.100 | 0.100 |
| Natuphos 5000AL (500 FTU) | 0.010 | 0.010 | 0.010 |
| *L*-lysine HCl | 0.293 | 0.256 | 0.210 |
| *DL*-methionine | 0.383 | 0.325 | 0.264 |
| *L*-threonine | 0.150 | 0.120 | 0.087 |
| *L*-valine | 0.140 | 0.104 | 0.064 |
| **Calculated nutrient composition, %** | | | |
| Dry matter | 87.8 | 87.8 | 87.7 |
| ME, MJ/kg | 12.30 | 12.55 | 12.80 |
| CP | 21.6 | 20.0 | 18.5 |
| Ether extract | 6.07 | 6.47 | 6.89 |
| Starch | 37.5 | 39.8 | 42.0 |
| Ash | 6.01 | 5.55 | 5.08 |
| Ca | 0.86 | 0.78 | 0.71 |
| P | 0.62 | 0.56 | 0.53 |
| Dig. P | 0.43 | 0.39 | 0.37 |
| Na+K-Cl, meq/100g | 27.0 | 25.5 | 22.0 |
| C18:2 | 3.02 | 2.95 | 2.87 |
| Lys | 1.41 | 1.27 | 1.14 |
| Dig. Lys | 1.22 | 1.10 | 0.98 |
| Dig. M+C/dig. Lys | 0.75 | 0.75 | 0.75 |
| Dig. Thr/dig. Lys | 0.67 | 0.67 | 0.67 |
| Dig. Val/dig. Lys | 0.80 | 0.80 | 0.80 |

* Premix providing per kg of diet: vitamin A (retinyl acetate), 10000 IU; vitamin D_3_ (cholecalciferol), 2500 IU (1-14 days) and 2000 IU (15-39 days); vitamin E (dl-α-tocopherol acetate), 50 mg; vitamin K_3_ (menadione), 1.5 mg; vitamin B_1_ (thiamine), 2.0 mg; vitamin B_2_ (riboflavin), 7.5 mg; niacin, 35 mg; D-pantothenic acid, 12 mg; vitamin B_6_ (pyridoxine-HCl), 3.5 mg; vitamin B_12_ (cyanocobalamine), 20 µg; folic acid, 1.0 mg; biotin, 0.2 mg; choline chloride, 460 mg; Fe (FeSO_4_.H_2_O), 80 mg; Cu (CuSO_4_.5H_2_O), 12 mg; Zn (ZnO), 60 mg; Mn (MnO), 85; I (Ca(IO_3_)_2_), 0.8 mg; Co (Co_2_CO_3_(OH)_2_), 0.77 mg; Se (Na_2_O_3_Se), 0.15 mg.

ME, metabolisable energy; CP, crude protein; Ca, calcium; P, phosphorus; Dig. Digestibility; Lys, Lysine; Na, sodium; K, potassium; Cl, chlorine; M+C, methionine+ *L*-cysteine; Thr, threonine; Val, valine.

**Table S2.** Analysis of diet composition (as-fed)

| Items | Starter diets | Grower diets | | Finisher diets | |
| --- | --- | --- | --- | --- | --- |
|  | Basal | Basal | Basal+25-OH-D_3_ | Basal diets | Basal+25-OH-D_3_ |
| DM, % | 88.5 | 88.2 | 88.5 | 88.3 | 88.2 |
| CP, % | 21.7 | 20.5 | 21.1 | 18.7 | 18.2 |
| Ash, % | 5.00 | 4.80 | 5.00 | 4.40 | 4.40 |
| Ca, % | 0.80 | 0.83 | 0.82 | 0.68 | 0.63 |
| P, % | 0.57 | 0.58 | 0.56 | 0.47 | 0.43 |
| Ether extract, % | 5.60 | 6.20 | 6.00 | 6.50 | 6.60 |
| Vitamin D_3_, IU/kg | 2020 | 1970 | 1890 | 1690 | 1650 |
| 25-OH-D_3_, μg/kg | <LOQ | <LOQ | 49.0 | <LOQ | 77.3 |

DM, dry matter; CP, crude protein; Ca, calcium; P, phosphorus; 25-OH-D_3_, 25-hydroxyvitamin D_3_.

**Table S3.** The primers for quantitative real-time PCR

| Gene | Gene ID | Primer | Sequence (5′-3′) | Size (bp) |
| --- | --- | --- | --- | --- |
| *NaPi-IIb* | NM_204474.2 | Reverse | tcatccatcatcgtcagcat | 81 |
|  |  | Forward | aatgtttgcccccataatga |  |
| *Calbindin-1* | NM_205513.1 | Reverse | aggcaggcttggacttaac | 97 |
|  |  | Forward | acctgagcaagctcaacgat |  |
| *VDR* | NM_205098.1 | Reverse | aagtcatcgacaccctcctg | 106 |
|  |  | Forward | atcctgctgctgaatttgct |  |
| *NaPi-IIa* | XM_015293844.2 | Reverse | cctcatcctcctggtcaaaa | 88 |
|  |  | Forward | tgggaggtcagtgttgatga |  |
| *Claudin-1* | NM_001013611.2 | Reverse | gtctttggtggcgtgatctt | 117 |
|  |  | Forward | tctggtgttaacgggtgtga |  |
| *ZO-1* | XM_015278981.2 | Reverse | ggtcagccagatgtggattt | 81 |
|  |  | Forward | ccgaagcattccatcttcat |  |
| *Mucin-2* | NM_001318434.1 | Reverse | tgccagcctttttatgctct | 80 |
|  |  | Forward | agtggccatggtttcttgtc |  |
| *IL-1β* | NM_204524.1 | Reverse | gtttttgagcccgtcacct | 117 |
|  |  | Forward | cacgaagcacttctggttga |  |
| *IL-6* | NM_204628.1 | Reverse | ctcctcgccaatctgaagtc | 100 |
|  |  | Forward | ccctcacggtcttctccata |  |
| *TNF-α* | NM_204267.1 | Reverse | agatgggaagggaatgaacc | 120 |
|  |  | Forward | actgggcggtcatagaacag |  |
| *TGF-1β* | NM_001318456.1 | Reverse | ctgtacaacagcacccagga | 88 |
|  |  | Forward | gctctttggcccaatactca |  |
| *Runx2* | NM_204128.1 | Reverse | caggcatgtcactgggtatg | 115 |
|  |  | Forward | tatggagtgctgctggtctg |  |
| *Phex* | NM_001199277.2 | Reverse | tgccaactatctggtgtgga | 100 |
|  |  | Forward | tccatggatcactcgtgaaa |  |
| *Dmp1* | NM_206993.1 | Reverse | agaggacagaagcgcagtgt | 80 |
|  |  | Forward | cctctgtctccaagctgtcc |  |
| *Sost* | XM_025144077.1 | Reverse | gacagaaatcatccccgaga | 83 |
|  |  | Forward | cctggttcatcgtgttgttg |  |
| Cathepsin K | NM_204971.2 | Reverse | atatgaccagcgaggaggtg | 88 |
|  |  | Forward | gggacgtacagagtgccatt |  |
| *V-ATPase* | NM_001293241.1 | Reverse | ggcttggtgaagaaatccaa | 97 |
|  |  | Forward | accgagcagtttccatgttc |  |
| *OPG* | XM_015283019.2 | Reverse | tgggacaaagatcagcacag | 106 |
|  |  | Forward | ctccttgttttgctgcttcc |  |
| *RANKL* | NM_001083361.1 | Reverse | gtccagcgtattctgggaaa | 116 |
|  |  | Forward | atgagatgggcaaaaggttg |  |
| *β-actin* | NM_205518.1 | Reverse | gctacagcttcaccaccaca | 90 |
|  |  | Forward | tctcctgctcgaaatccagt |  |
| *GAPDH* | NM_204305.1 | Reverse | tgggaagcttactggaatgg | 88 |
|  |  | Forward | cttggctggtttctccagac |  |

*NaPi-II,* sodium-dependent phosphorus transport protein II; *VDR,* vitamin D receptor; *ZO-1,* zonula occludens-1; *IL*, interleukin; *TNF-α*, tumor necrosis factor-alpha; *TGF-1β*, transforming growth factor beta 1; *Runx2*, runt related transcription factor 2; *Phex*, phosphate regulating endopeptidase homolog x-linked; *Dmp 1*, dentin matrix protein 1; *Sost*,Sclerostin; *V-ATPase*, V-type proton ATPase; *OPG*, osteoprotegerin; *RANKL*, Receptor activator of nuclear factor-κ B ligand; *GAPDH*, glyceraldehyde-3-phosphate dehydrogenase.
